# Gastrointestinal-focused enhanced recovery after surgery compliance and restoration of post-operative bowel function in gynecological malignancies: a prospective cohort study

**DOI:** 10.3389/fonc.2026.1793931

**Published:** 2026-03-12

**Authors:** Debasmita Saha, Arpitha Anantharaju, Veena P., Sakthirajan Panneerselvam, Anusuya R.

**Affiliations:** 1 Department of Obstetrics and Gynecology, Jawaharlal Institute of Postgraduate Medical Education and Research (JIPMER), Pondicherry, India; 2 Department of Anesthesiology and Critical Care, Jawaharlal Institute of Postgraduate Medical Education and Research (JIPMER), Pondicherry, India; 3 Department of Biostatistics, Jawaharlal Institute of Postgraduate Medical Education and Research (JIPMER), Pondicherry, India

**Keywords:** compliance, enhanced recovery after surgery, ERAS, gastrointestinal, bowel, gynecological malignancies, oral tolerance, quality of recovery

## Abstract

**Background:**

Restoring post-operative gastrointestinal (GI) function is key to overall recovery. While data on enhanced recovery after surgery (ERAS) compliance in gynecological oncology exist, the specific impact of GI-focused components on early bowel function remains understudied.

**Objectives:**

The primary objective was to evaluate the rate of oral fluid tolerance at 6 h post-operatively in patients undergoing elective laparotomy for gynecological malignancies and determine the impact of GI-focused ERAS compliance on this outcome. Secondary objectives included evaluating post-operative complications, length of stay (LOS), and readmissions.

**Methods:**

This prospective cohort study was conducted at a tertiary cancer center in India (February 2023–November 2024) with 165 patients. Fifteen GI-focused ERAS components were selected. Compliance was calculated as the percentage of components fulfilled per patient. The association between compliance and early oral tolerance was analyzed using logistic regression and receiver operating characteristic (ROC) analysis.

**Results:**

The median compliance rate was 80% [interquartile range (IQR) 73.3%–86.6%]. Early oral tolerance was achieved in 56.4% of patients. On multivariate analysis, while individual components showed variation, overall high compliance was positively associated with early oral tolerance. ROC analysis identified a compliance threshold of 79% (AUC 0.749; sensitivity 60.2%, specificity 83.3%) as a significant predictor for early oral tolerance. However, higher compliance did not significantly reduce LOS or major complications in this cohort.

**Conclusion:**

A compliance threshold of >79% in GI-focused ERAS components is associated with improved early oral tolerance in open gynecological oncology surgeries. While this did not translate to reduced LOS, likely due to non-medical discharge factors, targeting high compliance facilitates early physiological recovery.

## Introduction

Gynecologic oncology surgeries are one of the most performed surgeries worldwide and in India. Gynecologic oncology surgeries pose a significant risk of post-operative stress and morbidity by particularly affecting gastrointestinal (GI) recovery (1). Paralytic ileus is a common GI complication following these surgeries, characterized by impaired intestinal motility, particularly after extensive cytoreductive procedures. This can lead to various complications and is a primary factor influencing post-surgical hospital stay and recovery, while imposing economic burden. Hence, restoring the post-operative GI function is key to overall post-operative recovery, early discharge, and patient wellbeing. Consequently, facilitating a faster recovery following cytoreductive surgeries is of utmost importance, as it minimizes the time between the surgical intervention and functional recovery. Enhanced recovery after surgery (ERAS) protocol has fundamentally changed perioperative care by integrating evidence-based practices, designed to optimize surgical outcomes and improve patient recovery processes. The concept of ERAS encompasses a comprehensive set of peri-operative interventions, aimed at reducing surgical stress on patients. This multi-modal approach integrates various aspects, including physical rehabilitation, nutritional management, anesthesia protocols, and surgical techniques.

Numerous randomized controlled trials (RCTs) have consistently shown that the ERAS protocol is associated with significant improvement in post-operative quality of life, reduced hospital stay, and greater cost-effectiveness, without increasing post-operative morbidity in various surgical fields (2). Most of the evidence for ERAS comes predominantly from studies on colorectal surgeries. Patients with ovarian cancer often experience GI symptoms, such as abdominal bloating and delayed gastric emptying, which may not occur in patients with colorectal cancer, whose disease is typically more localized and hence presents with localized symptoms. Gynecologic oncology surgeries are markedly different from those in other surgical fields, as patients frequently present with advanced disease that requires complex surgical procedures, including upper abdominal procedures (3). Surgeons may frequently need to do extensive surgery to optimally debulk upper abdominal disease, which will influence post-operative recovery. Because of such extensive surgeries and surgeon’s preferences, all the post-operative ERAS components might not be complied with, reducing the overall ERAS compliance rate.

While the ERAS protocol has been well recognized in gynecological oncology surgeries, the impact of protocol compliance rate on post-operative outcomes remains under-explored. This study investigated the effectiveness of GI-focused ERAS components on post-operative GI recovery outcomes in gynecological oncology surgeries. The GI-focused key pre-operative, intra-operative, and post-operative components of ERAS were segregated to tailor them for patients with gynecological malignancies with the objective of expediting GI recovery.

While length of stay (LOS) is a traditional metric for ERAS success, it is often confounded by non-medical factors such as social support and logistics, particularly in resource-limited settings. Conversely, oral fluid tolerance at 6 h represents a direct, patient-centered measure of physiological GI recovery. It serves as an early surrogate for the resolution of gastric ileus and a necessary precursor to full functional recovery, making it a valuable immediate marker for surgical quality auditing.

## Materials and methods

**Figure 1:**
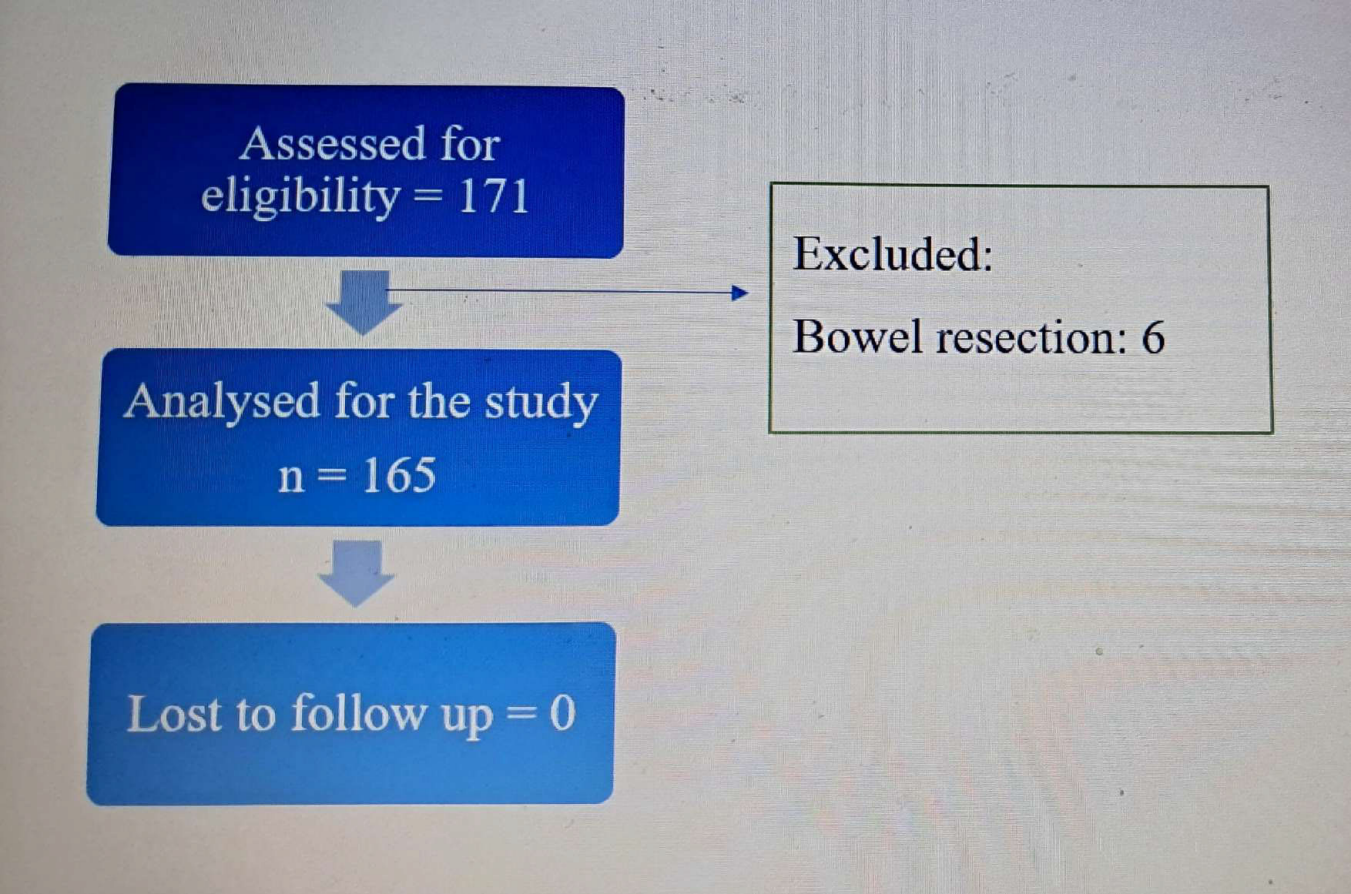
Flowchart of the study depicting participant enrollment and analysis.

This prospective cohort study was conducted from February 2023 to November 2024 with 165 patients. **Figure 1** shows the flowchart of the study depicting participant enrollment and analysis. This study was approved by Jawaharlal Institute of Postgraduate Medical Education and Research Institutional Ethics Committee (IEC) Interventional Studies CDSCO Reg No. ECR/342/Inst/PY/2013/RR-19 on 13 March 2023. All methods were carried out in accordance with relevant guidelines and regulations.

Inclusion criteria were women with ovarian, endometrial, or cervical carcinoma planned for elective open staging surgery, primary cytoreduction, interval cytoreduction, or secondary cytoreduction. Exclusion criteria were surgery involving bowel resection anastomosis or stoma placement (since extensive retroperitoneal dissection and cytoreduction even without bowel resection can cause significant autonomic dysfunction leading to ileus) and hyperthermic intraperitoneal chemotherapy (HIPEC).

Demographic, clinical, and surgical variables of the study population were recorded. GI-focused ERAS components, including five each from pre-operative, intra-operative, and post-operative components are shown in **Table 1**.

**Table 1:** GI-focused ERAS components.

Pre-operative	Intra-operative	Post-operative
No mechanical bowel preparation	Opioid-sparing multimodal analgesia	Removal of Nasogastric (NG) tube at extubation
Minimal fasting for solids (< 6 hours)	Goal-directed fluid therapy	Opioid-sparing analgesia
Minimal fasting for liquids (< 2 hours)	Maintenance of normothermia	Chewing gum
Opioid-sparing pre-medication	PONV prophylaxis	Early ambulation
Complex carbohydrate loading	Avoidance of IP drain	Early removal of catheter and drain

Post-operative complications were graded by the Clavien–Dindo classification as minor (grades 1 and 2) and major (grades 3, 4, and 5). Quality of recovery was measured by the QoR-15 (15-item quality of recovery) score. All patients were asked to complete a QoR-15 questionnaire, under the guidance of the investigator at the 72-h post-operative period.

Standard general anesthesia was induced and maintained according to institutional protocol. Each individual component was implemented for every patient, and the corresponding outcome was collected in a prospective manner with the assistance of nursing staff and anesthesiologists. Compliance was assessed as a binary variable (1 = fulfilled, 0 = not fulfilled) for each of the 15 selected GI-focused components. “Fulfilment” was defined strictly according to the ERAS society guidelines (e.g., “Early feeding” was defined as solid food intake within 24 h). Total compliance was calculated as the percentage of items successfully implemented out of the total applicable items for that patient, designated as (*n*). For patients with diabetes, the total number of GI-focused ERAS components was taken as (*n* − 1), as the pre-operative component of pre-carbohydrate loading was excluded. In the case of type 3 radical hysterectomy for carcinoma cervix, the total number of ERAS components was also taken as (*n* − 1), given that the removal of the urinary catheter required a period exceeding the 24-h post-operative period. Each fulfilled ERAS component was assigned a point value of one, thereby expressing the rate of compliance as a percentage. Patients were eligible for discharge when they fulfilled the criteria, which included mobilization (>6 h daily), tolerance to a regular diet, adequate pain relief with oral analgesics (VAS < 40 of 100), normal urination and bowel movements, and absence of any signs of post-operative complications. Subsequent to discharge, patients were monitored for potential readmissions due to post-operative complications within a 30-day period following surgery, through either outpatient or emergency department services.

The primary outcome measure was the tolerance of the initial oral fluid administered at the 6-h post-operative period. Upon recovery from anesthesia at the 6-h post-operative period, patients were offered 50 mL of clear fluid while in a reclined position, remaining in this position for a duration of 10 to 20 min. Tolerance was defined as no nausea or vomiting within 2 h. If intolerance occurred, patients were offered fluids again after 4 h; if still not tolerated, patients were reassessed the next morning. If symptoms continued, paralytic ileus was suspected. Patients were followed until 30 days post-discharge. Our secondary outcome variables were as follows: quality of recovery (QoR-15 score), time to passage of flatus, time to passage of stools, length of hospital stay, post-operative complications, readmissions, and reoperations. Sample size was calculated by anticipating oral tolerance as 70% with 10% relative precision and 5% level of significance. A total of 165 cases were recruited.

### Statistical analysis

Categorical data are presented as frequency and percentage, while continuous data are presented as mean with standard deviation or median with (minimum, maximum), depending on the distribution of data. The normality of distribution of data was checked using the Kolmogorov–Smirnov test.

Continuous variables were compared using independent Student’s *t*-test or the Mann–Whitney *U* test based on normality distribution, while categorical variables were analyzed using the chi-square test or Fisher’s exact test as appropriate based on expected cell counts. Correlations were evaluated using Pearson’s or Spearman’s correlation based on the distribution of the data. Components of ERAS were initially analyzed through Fisher’s exact test, with significant components (*p* < 0.05) included in multiple logistic regression analysis. The optimal cutoff value for compliance was determined using the Youden Index (*J* = sensitivity + specificity − 1) to maximize the discriminatory power of the model. All statistical analyses were conducted using SPSS version 27, with a significance level set at 5%.

## Results

A total of 165 cases were analyzed, with no loss to follow-up. Baseline characteristics of the study population are shown in **Table 2**.

**Table 2:** Characteristics of the study population.

Characteristics	Categories	Percentage
Comorbidities	Diabetes	33.9
	Hypertension	29.1
Carcinoma	Ovary	61.8
	Endometrium	34.5
	Cervix	3.6
Stage of malignancy	1	77.6
	2,3	18.2
	4	4.2
Post-operative complications	Minor	93.9
	Major	6.1

In our study, median age was 53 years (45, 60) and mean body mass index (BMI) was 26.1 (3.2). Diabetes was the most common comorbidity (33.9%). Most surgeries were performed for ovarian carcinoma (61.8%), and the majority of patients belonged to stage 1 of the disease (77.6%). Major complications (Clavien–Dindo grades 3 and 4) were reported in 6.1% of patients. The median ERAS compliance rate in our study was 85% (57, 100). A compliance rate of 100% was achieved in 28 patients (16.9%). The median time to passage of flatus and stools was 24 h (24, 48) and 48 h (48, 72) respectively.

The rate of oral tolerance to clear fluids at the 6-h post-operative period was 93.9% (155 patients). Of these 155 patients, 150 (96.8%) did not fail to tolerate solid diet the next day.

**Figure 2:**
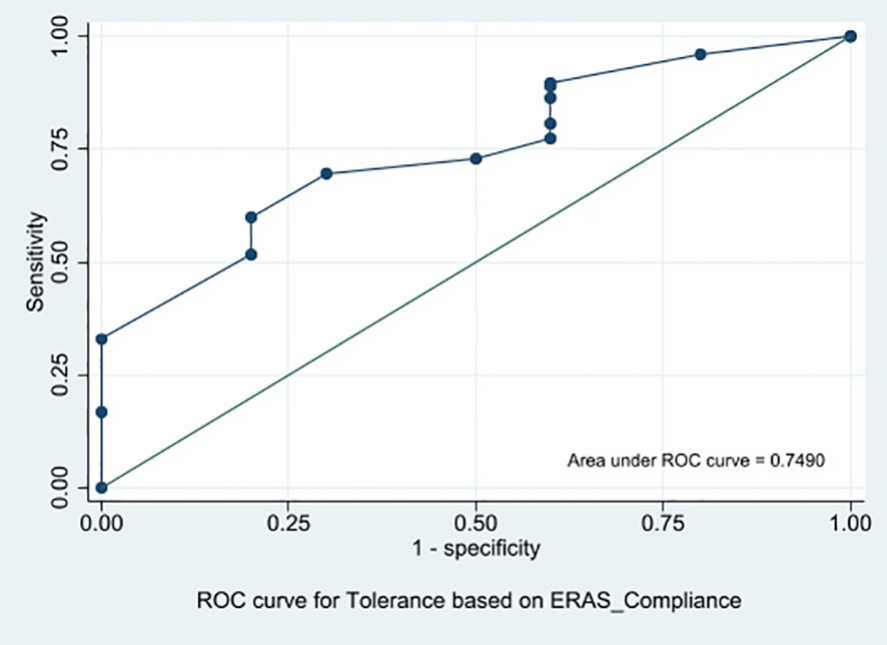
ROC showing association of oral tolerance with gastrointestinal-focused ERAS compliance rate.

Association of oral tolerance with GI-focused ERAS compliance rate was significant (*p* = 0.008). The compliance rate above which this oral tolerance was significantly increased for all patients, as determined from ROC (AUC 0.749), was 79%, with 69.9% sensitivity and 70% specificity (95% CI 61%–88%), as shown in **Figure 2**.

**Table 3** shows the association of GI-focused ERAS compliance rate with post-operative parameters. The association of QoR-15 score at the 72-h post-operative period with compliance rate, as determined by Spearman’s correlation, was significant (*p* < 0.001, 95% CI 0.158–0.447). The association of compliance with time to passage of flatus and stools was significant (*p* < 0.001), but the association of compliance with length of hospital stay was not significant (*p* = 0.442).

**Table 3:** Association of GI-focused ERAS compliance rate with post-operative parameters.

Parameters	Median (min, max)	P value
Quality of recovery (QOR-15) score	113 (90, 127)**	< 0.001^*^
Time to passage of flatus	24 (24, 48)	< 0.001^*^
Time to passage of stools	48 (24, 48)	< 0.001^*^
Length of hospital stay	8 (5, 12.5)	0.442^*^

* - Spearman’s correlation.

**- total score: 150.

The association of ERAS compliance with major post-operative complications (0.291), readmission (*p* = 0.222), and reoperation (*p* = 0.927) was not statistically significant, as determined by the Mann–Whitney *U* test. However, this study was underpowered to detect significant differences in major complications due to the low overall incidence rate (6.1%). Similarly, this study was underpowered to detect significant differences in readmissions and reoperations due to the low overall incidence rate (3% each).

**Table 4** shows the association of GI-focused ERAS components with oral tolerance to clear fluids at the 6-h post-operative period. Goal-directed fluid therapy and chewing gum were found to be independently and significantly associated with the achievement of oral tolerance within 6 h of the post-operative period. However, multivariate logistic regression analysis indicates that individual compliance with any of the components does not predict the incidence of oral tolerance. There was no significant association between oral tolerance and type of malignancy (*p* = 0.656), stage of malignancy (*p* = 0.081), operating surgeon (*p* = 0.058), and type of surgery (*p* = 0.058).

**Table 4:** Association of GI-focused ERAS components with oral tolerance to clear fluids at 6 hours postoperative period.

GI bundle of ERAS components	Individual compliance rate (%)	p value*	p value**
Mechanical bowel preparation	50.3	0.057	
Pre-operative fasting for solids	100	NA	
Pre-operative fasting for liquids	92.1	0.184	
Pre-operative complex carbohydrate loading	68.8	0.649	
Opioid sparing premedication	100	NA	
Avoidance of drain	79.3	0.432	
Intraoperative multimodal analgesia	99.3	0.063	
Goal-directed fluid therapy	52.1	0.018	0.061
Maintenance of normothermia	100	NA	
PONV prophylaxis	100	NA	
Removal of Nasogastric (NG) tube at extubation	100	NA	
Postoperative multimodal analgesia	100	NA	
Early removal of drain	9.7	1	
Early removal of urinary catheter	80.5	0.121	
Chewing gum	55.2	0.023	0.125

*Fischer’s exact test.

**Multiple logistic regression analysis.

NA- indicates variables with 100% compliance (zero variance), which were excluded from statistical correlation testing.

PONV – postoperative nausea and vomiting.

## Discussion

The median GI-focused ERAS compliance rate in our study is 85% (57, 100). An ERAS compliance rate of 100% is achieved in 28 patients (16.9%). The higher compliance rate with the GI-focused ERAS protocol is significantly associated with improved oral tolerance, enhanced quality of recovery, and a shorter time to the passage of flatus and stool, without any significant difference in major post-operative complications, readmissions, and reoperations; length of hospital stay is reduced. The rate of oral tolerance at the 6-h post-operative period in our study population after the introduction of the GI-focused ERAS protocol is 93.9%. ERAS compliance rate above 79% is a significant predictive cutoff for better oral tolerance. We identified a compliance threshold of 79%. It is important to note that this figure is derived from our specific cohort and should be interpreted as a clinical benchmark rather than an absolute cutoff. It suggests that “high adherence” (roughly four out of five interventions) is necessary to see physiological benefits.

To the best of our knowledge, no prior studies have linked ERAS compliance rates to oral tolerance and quality of recovery in post-operative gynecological onco-surgery patients. In a retrospective study on open radical hysterectomy, the median ERAS compliance was 70% [interquartile range (IQR) 65%–75%] (4). In a meta-analysis by Sauro et al., mean compliance was 74.7% (10.2%) (5). Pache et al. showed that the mean ERAS compliance rate among patients with complications was 76% (17%) and that among patients without complications was 84% (12%) (*p* < 0.001) (6).

Our study showed a significant increase in QoR-15 score with a higher ERAS compliance rate (*p* < 0.001). Ferrari et al. showed a significant increase in QoR-15 score with the ERAS protocol after hysterectomy (including both benign and malignant cases) on post-operative day 1 (*p* < 0.001) and on the day of discharge (*p* < 0.001) (7). Shen et al. observed that the ERAS compliance rate exceeding 80% on patients undergoing hysterectomy for benign conditions was associated with higher QoR-15 scores (*p* < 0.001) (8).

The rate of major complications in our study was 6.1%, which is much lower compared to the 10.2% reported by Iniesta et al. (9) and 26.2% by Pache et al. (6). Our study demonstrated no significant association between ERAS compliance rate and major post-operative complications, but the comparison was not reliable. These complications were predominantly wound infections and burst abdomen, which are mainly due to surgical procedure, and not related to GI motility. Iniesta et al. showed that a compliance rate of 80% or above experienced significantly fewer complications (*p* < 0.001) (9). We had an overall ERAS compliance rate of >80%, and compliance was not normally distributed across the groups; thus, sub-group analysis of compliance rate with complications is not reliable.

Our study demonstrated no statistically significant correlation between ERAS compliance and length of hospital stay. In an Indian tertiary care setting, discharge is often delayed for social/logistic reasons (long travel distance, fear of complications at home, stay until suture removal or until histopathology report is obtained, and family issues) rather than purely medical readiness. In an RCT on ovarian carcinoma, it was observed that higher ERAS compliance was linked to a shorter median LOS (10). The RACCE study reported that the median length of hospital stay reduced significantly with ERAS (11). The PROFAST study indicated a reduction of median length of hospital stay by 2 days with ERAS (3). A recent meta-analysis conducted by Bisch et al. in gynecologic oncology reported a mean reduction in the LOS of 1.64 days in the ERAS group (95% CI 1.18–2.10 days) as compared to the control group (12). Ferrari et al., in their study involving both benign and malignant gynecological cases, also showed that the LOS was significantly lower in the ERAS group compared to the control group (*p* < 0.001) (7). Iniesta et al. in the study on open gynecological surgeries (both benign and malignant) showed that those with a compliance rate of 80% or above experienced significantly reduced length of hospital stay (*p* < 0.001) (9). Interestingly, improved GI recovery did not statistically shorten the LOS. In our setting (a tertiary public referral center), discharge is frequently delayed by logistical factors, such as arranging transport for patients from remote locations, rather than medical necessity. Thus, early oral tolerance may be a more accurate reflection of the biological efficacy of the ERAS protocol than LOS in this specific demographic.

Our study demonstrated that higher ERAS compliance was associated with a shorter time to passage of flatus (*p* < 0.001) and stools (*p* < 0.001). Ferrari et al. showed significant reduction in the time to passage of flatus and stools with the ERAS protocol (7). In the study conducted by Suresh et al. in patients undergoing laparotomy for proven or suspected gynecological malignancy, the ERAS group had an early return of bowel function (28.17 h vs. 32 h, *p* = 0.0021), as compared to the standard care group, with no significant difference in other post-operative complications and readmission rates (0.72% vs. 2.12%, *p* = 0.624) (13). Lindemann et al. showed that the implementation of ERAS reduced the length of hospital stay (*p* = 0.026), median fasting time for solids (13.1 h post-ERAS vs. 16 h pre-ERAS; *p* < 0.001), and fluids (3.7 h vs. 11 h; *p* < 0.001), with no difference in readmission rates (14). Our study showed that there was no statistically significant correlation between ERAS compliance rate and readmission and reoperation. These results are similar to the RACCE study, which also reported no significant difference in the readmission rates (11). In a study on patients undergoing open gynecological surgeries (both benign and malignant cases), Iniesta et al. revealed that those with a compliance rate of 80% had no observed differences in readmission (*p* = 0.182) or reoperation rates (*p* = 0.078) (9).

Sub-analysis showed that individual compliance with chewing gum significantly improves oral tolerance. Since chewing gum is a low-cost, low-risk intervention, this is important for resource-limited setting even though multivariate analysis does not show any significant results. A meta-analysis showed that gum-chewing was significantly associated with lower rate of post-operative ileus (*p* = 0.0006), shorter time to first flatus (*p* = 0.0006), first bowel movement (*p* = 0.02), and shorter length of hospital stay (*p* < 0.00001) (15).

### Strengths and limitations

The major strength of this study lies in its prospective design conducted at a high-volume tertiary care institution, ensuring a diverse patient sample. It establishes a cutoff for GI-focused ERAS compliance rate predictive of improved GI function and hence functional recovery in the post-operative period in patients undergoing gynecologic oncology surgeries, providing a quantifiable target for clinical practice. Thus, it may help to reduce time to initiating adjuvant therapy post-surgery to achieve optimal oncological outcomes. The inclusion of patient-reported outcomes further enhances the comprehensiveness of recovery assessment. Our sub-analysis showed that individual compliance with chewing gum significantly improves oral tolerance. Since chewing gum is a low-cost, low-risk intervention, this is important for resource-limited setting.

However, complete adherence to all ERAS components was challenging due to surgical complexity, inter-surgeon variability, and the need for prolonged drain placement following extensive cytoreduction. The inclusion of patients with different types and stages of malignancy introduced variability in a surgical extent. Moreover, the skewed data distribution and high median compliance rate (85%) may have limited the robustness of associations with complications, readmissions, and reoperations. The study also did not reassess the correlation between ERAS compliance and the interval to initiation of adjuvant chemotherapy, which may hold greater oncologic significance than post-operative hospital stay.

## Conclusion

Our study showed that higher compliance to the GI-focused ERAS components is essential for faster post-operative recovery of GI function and the overall better quality of recovery in gynecological oncology surgeries. Surgeons should target more than 79% ERAS compliance to ensure optimal GI recovery. Moreover, increased compliance with the ERAS protocol does not lead to a significant change in major post-operative complications, readmissions, or reoperations. The length of hospital stay is reduced, though not significant. Sub-analysis showed that individual compliance with goal-directed fluid therapy and chewing gum significantly improves oral tolerance, but multiple logistic regression did not show any significant results. Our study suggests that compliance to all the GI-focused ERAS components as a whole is the best approach. Future prospective and randomized multicenter studies with larger sample sizes and strict adherence monitoring are needed to validate these results.

## Data Availability

The raw data supporting the conclusions of this article will be made available by the authors, without undue reservation.

## Glossary

1. Time to tolerate clear fluids—defined as the time from recovery from anesthesia until the patient tolerated intake of clear fluids, without vomiting or significant nausea within 2 h after meal, expressed in hours.

2. Time to passage of flatus—defined as the time from recovery from anesthesia, until the patient passed first flatus, expressed in hours.

3. Time to passage of stools—defined as the time from recovery from anesthesia, until the patient passed first stools, expressed in hours.

4. Length of hospital stay—defined as the number of nights spent in the hospital post-surgery, i.e., time from the day of surgery to the day of discharge, expressed in days.

5. Post-operative complications—defined as complications occurring during the hospital stay or any readmissions within 30 days after surgery by the guidelines for European perioperative clinical outcome definitions, graded by the Clavien–Dindo classification.

6. Quality of recovery—measured by the QoR-15 score.
